# A Case of an Intrathoracic Accessory Rib Revealed on Chest X-ray

**DOI:** 10.7759/cureus.75812

**Published:** 2024-12-16

**Authors:** Hany Abdelmasih, Moustafa Elramlawy, Marcelle Yakoub, Mina Zekry, Ahmed H Abdellatif

**Affiliations:** 1 Acute Medicine, Stoke Mandeville Hospital, Aylesbury, GBR; 2 Accident and Emergency, Stoke Mandeville Hospital, Aylesbury, GBR; 3 General Practice, Assiut University, Assiut, EGY; 4 Trauma and Orthopaedics, East Kent Hospitals University NHS Foundation Trust, Margate, GBR; 5 Medicine for Older People, Stoke Mandeville Hospital, Aylesbury, GBR

**Keywords:** accessory rib, anomaly, chest, intrathoracic, radiology

## Abstract

Accessory ribs are rare anatomical variations, typically cervical or lumbar, with intrathoracic accessory ribs being particularly uncommon. These anomalies are often asymptomatic but can cause issues like thoracic outlet syndrome. This case report describes a 36-year-old woman who was incidentally found to have an intrathoracic accessory rib on a chest X-ray. Conservative management was advised as the rib did not affect any vital structures and the patient was asymptomatic. The case highlights the importance of recognizing rare anomalies through imaging to avoid unnecessary workup.

## Introduction

Intrathoracic accessory ribs are rare anatomical anomalies characterized by an additional rib structure within the thoracic cavity. Unlike the more commonly observed cervical or lumbar accessory ribs, intrathoracic accessory ribs are exceedingly uncommon and often pose unique diagnostic challenges due to their atypical location. These ribs are usually asymptomatic and are often discovered incidentally during imaging for unrelated conditions, yet their proximity to vital structures within the thorax can occasionally result in complications. Depending on their size and orientation, intrathoracic accessory ribs may cause issues such as compression of neurovascular structures, leading to symptoms that mimic other thoracic conditions [1].

This case report presents a patient with an intrathoracic accessory rib, detailing the clinical presentation, imaging findings, and management approach. Through our case, we emphasize the importance of considering accessory intrathoracic ribs (though a rare anomaly) in the differential diagnosis to avoid unnecessary and excessive workup.

## Case presentation

**Figure 1:**
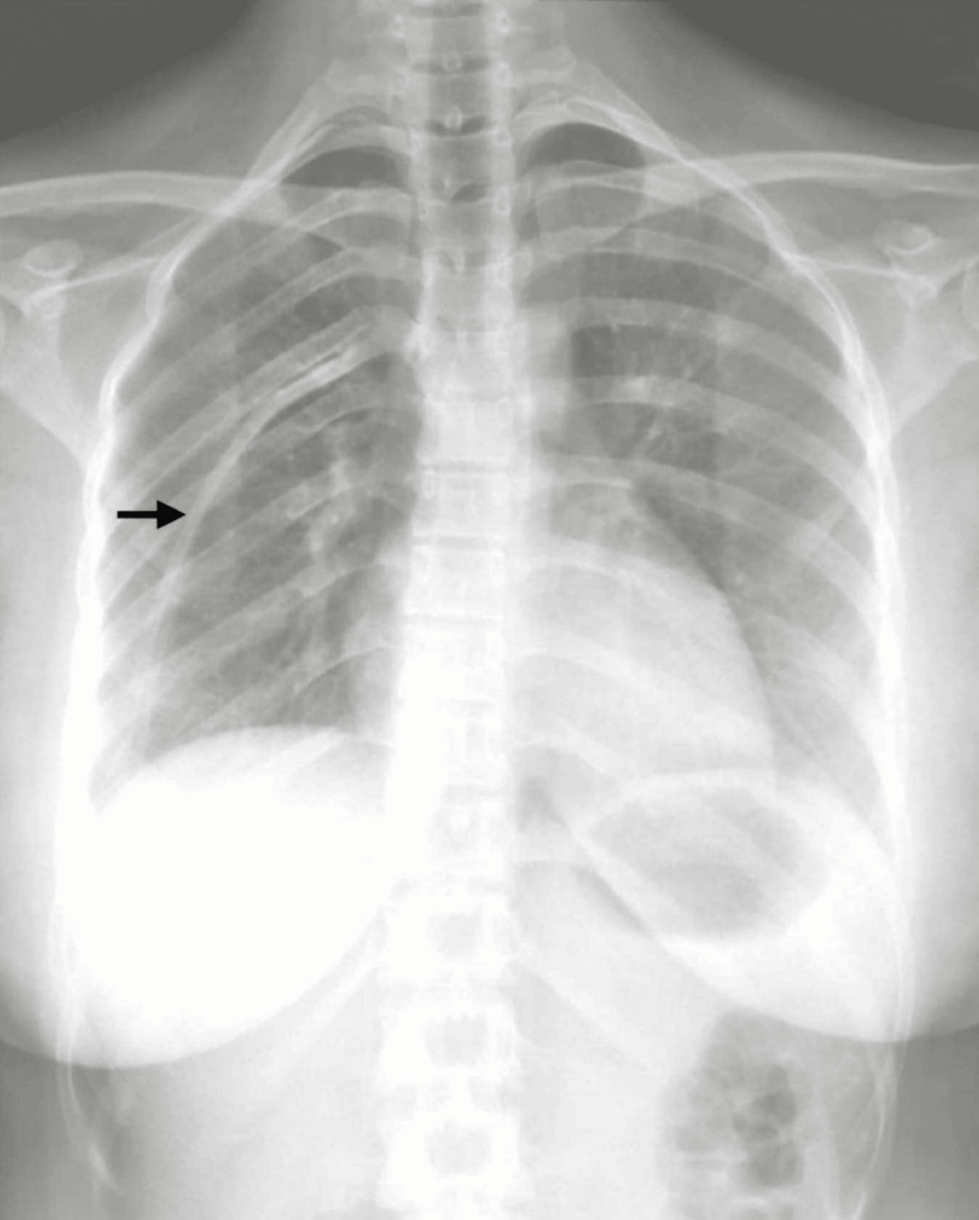
Fifth intrathoracic accessory rib in the right hemithorax.

**Figure 2:**
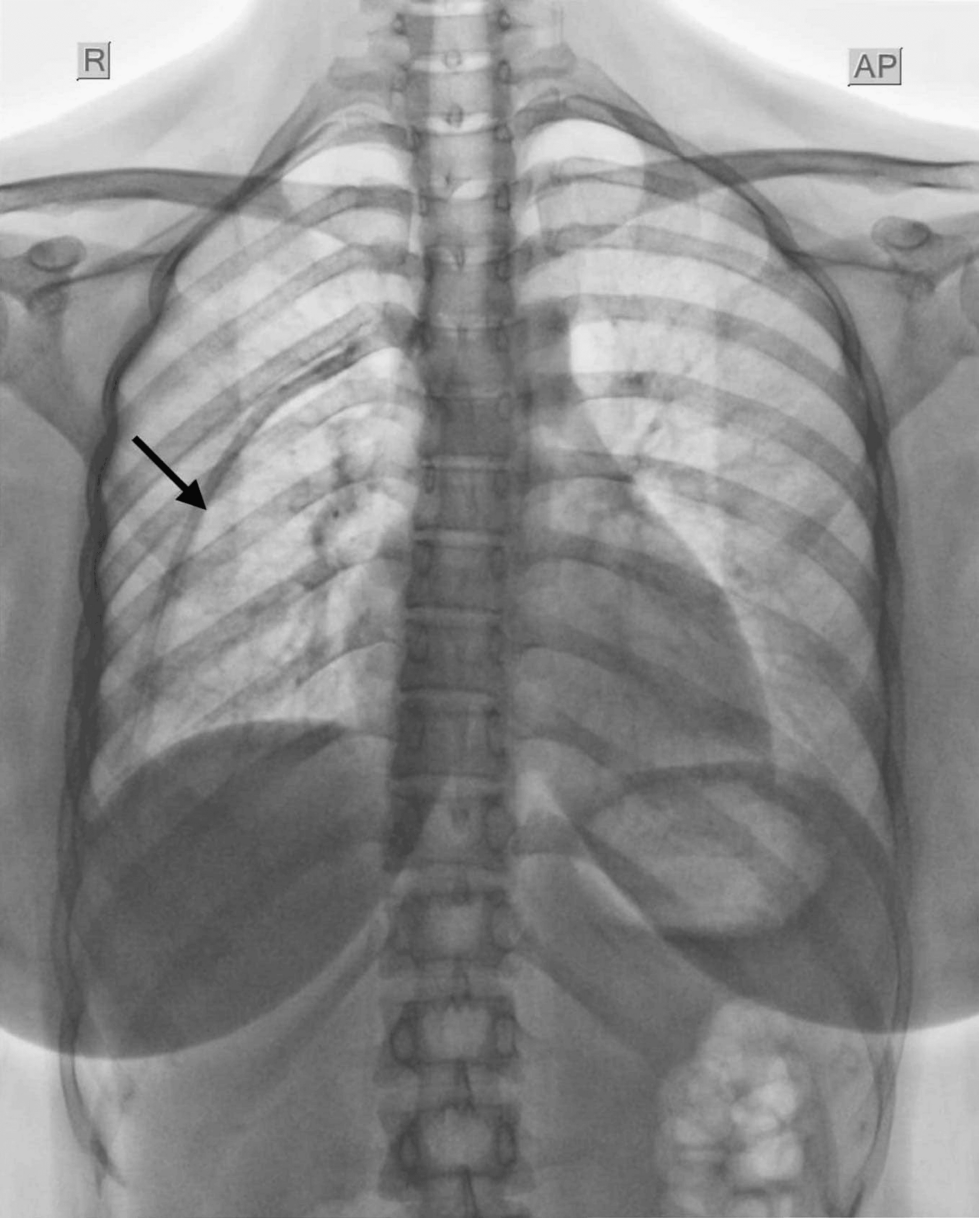
An enhanced chest radiograph showing evidence of a bifid intrathoracic fifth rib in the right hemithorax.

A 36-year-old Asian woman presented for a routine pre-employment health check with no significant past medical history. She reported no symptoms related to her respiratory or cardiovascular systems. The patient’s physical examination was normal, with no abnormalities detected in the respiratory, cardiovascular, or musculoskeletal systems. A routine chest X-ray (Figures 1, 2) was performed, which unexpectedly revealed an accessory rib extending downward from the right fifth rib and protruding into the thoracic cavity. The rib did not appear to impinge on any major thoracic structures, including the lungs, heart, or major blood vessels. Given the absence of symptoms and the lack of impingement on critical structures, conservative management was chosen. The patient was informed about the anomaly, reassured, and advised that no further workup was necessary unless symptoms developed.

## Discussion

Overview of accessory ribs

Accessory ribs are congenital anomalies that result from the persistence and subsequent ossification of embryonic costal processes. Most accessory ribs are cervical and often associated with thoracic outlet syndrome, while thoracic and lumbar accessory ribs are rarer, with intrathoracic accessory ribs being especially uncommon. These anomalies are typically detected incidentally during imaging for unrelated conditions, as in this case.

Clinical significance of intrathoracic accessory ribs

Intrathoracic accessory ribs are rare and frequently asymptomatic. However, depending on their size, location, and proximity to nearby structures, they can sometimes cause clinical symptoms. Complications may include thoracic outlet syndrome, where the accessory rib compresses neurovascular structures, or respiratory symptoms if the rib impinges on lung tissue.

The literature on intrathoracic ribs, a rare congenital anomaly, includes case reports that highlight the diagnostic challenges and clinical implications associated with these conditions. Apaydin et al. described a third accessory intrathoracic rib on the right side, emphasizing the importance of recognizing such anatomical variations to avoid misdiagnosis and ensure proper treatment [2]. Xue et al. expanded on this by proposing a classification system for intrathoracic ribs, offering a comprehensive review of the existing literature and discussing the potential complications these anomalies can cause, which may influence surgical planning [1]. Abdollahifar et al. highlighted the role of computed tomography (CT) in diagnosing rare rib anomalies, underscoring its importance in clinical practice for accurate identification and management [3]. Mahajan et al. reported a particularly complex case involving second and third bifid intrathoracic ribs, accompanied by block vertebrae and hypoplastic left lung, illustrating the need for thorough imaging to evaluate associated congenital abnormalities [4]. A case reported by Kamano et al. described a rare instance of a bifid intrathoracic rib and proposed a classification system for intrathoracic ribs, categorizing them based on their anatomical characteristics [5]. Muise et al. described a rare congenital anomaly involving a supernumerary intrathoracic rib in a child [6].

Although accessory ribs are typically asymptomatic, they can occasionally lead to complications such as localized pain or even lung collapse, as seen in cases of bifid ribs and intrathoracic ribs in pediatric patients [7,8].

## Conclusions

This case underscores the importance of thorough imaging and clinical assessment in detecting rare congenital anomalies, such as intrathoracic accessory ribs. Although often asymptomatic, these anomalies can have clinical significance and may require intervention based on the presence of symptoms and potential complications. Patient education is an essential component in the management of these cases to avoid unnecessary investigations.

## References

[1] Xue X, Zhao S, Li K, Zhao B (2021). Intrathoracic rib: rare rib anomaly, review of the literature and proposal for classification. Int J Med Sci.

[2] Apaydin M, Sarsilmaz A, Varer M (2009). Third accessory (supernumerary) intrathoracic right rib. Surg Radiol Anat.

[3] Abdollahifar MA, Abdi S, Bayat M, Masteri Farahani R, Abbaszadeh HA (2017). Recognition of a rare intrathoracic rib with computed tomography: a case report. Anat Cell Biol.

[4] Mahajan PS, Hasan IA, Ahamad N, Al Moosawi NM (2013). A unique case of left second supernumerary and left third bifid intrathoracic ribs with block vertebrae and hypoplastic left lung. Case Rep Radiol.

[5] Kamano H, Ishihama T, Ishihama H, Kubota Y, Tanaka T, Satoh K (2006). Bifid intrathoracic rib: a case report and classification of intrathoracic ribs. Intern Med.

[6] Muise ED, Lee EY, Paltiel HJ, Gaffin JM (2020). Supernumerary intrathoracic rib, a rare congenital anomaly: case report and review of the literature. Pediatr Pulmonol.

[7] Basarslan F, Bayarogulları H, Tutanc M, Arica V, Yilmaz C, Davran R (2012). Intrathoracic rib associated with pulmonary collapse in a pediatric patient. Iran J Radiol.

[8] Batra D, Lawner BJ (2006). Bifid fifth rib in a 9-year-old girl with chest pain. J Am Osteopath Assoc.

